# Comparative In Vitro and In Silico Analysis of the Selectivity of Indirubin as a Human Ah Receptor Agonist

**DOI:** 10.3390/ijms19092692

**Published:** 2018-09-10

**Authors:** Samantha C. Faber, Anatoly A. Soshilov, Sara Giani Tagliabue, Laura Bonati, Michael S. Denison

**Affiliations:** 1 Department of Environmental Toxicology, University of California, Davis, CA 95616, USA; scfaber@unc.edu (S.C.F.); soshilov@gmail.com (A.A.S.); 2 Department of Earth and Environmental Sciences, University of Milano-Bicocca, Milan 20126, Italy; s.gianitagliabue@campus.unimib.it (S.G.T.); laura.bonati@unimib.it (L.B.)

**Keywords:** Ah receptor, AhR, indirubin, TCDD, in vitro, in silico

## Abstract

The aryl hydrocarbon receptor (AhR) is a ligand-dependent transcription factor that modulates gene expression following its binding and activation by structurally diverse chemicals. Species differences in AhR functionality have been observed, with the mouse AhR (mAhR) and human AhR (hAhR) exhibiting significant differences in ligand binding, coactivator recruitment, gene expression and response. While the AhR agonist indirubin (IR) is a more potent activator of hAhR-dependent gene expression than the prototypical ligand 2,3,7,8-tetrachlorodibenzo-p-dioxin (TCDD), it is a significantly less potent activator of the mAhR. DNA binding analysis confirmed the greater potency/efficacy of IR in stimulating transformation/DNA binding of the hAhR in vitro and domain-swapping experiments demonstrated that the enhanced response to IR was primarily due to the hAhR ligand binding domain (LBD). Site-directed mutagenesis and functional analysis studies revealed that mutation of H326 and A349 in the mAhR LBD to the corresponding residues in the hAhR LBD significantly increased the potency of IR. Since these mutations had no significant effect on ligand binding, these residues likely contribute to an enhanced efficiency of transformation/DNA binding by IR-bound hAhR. Molecular docking to mAhR LBD homology models further elucidated the different roles of the A375V mutation in TCDD and IR binding, as revealed by [^3^H]TCDD competitive binding results. These results demonstrate the differential binding of structurally diverse ligands within the LBD of a given AhR and confirm that amino acid differences within the LBD of AhRs contribute to significant species differences in ligand response.

## 1. Introduction

The aryl hydrocarbon receptor (AhR) is a basic helix-loop-helix Per-ARNT-Sim (bHLH-PAS) ligand-dependent transcription factor that mediates the induction/repression of a large battery of genes involved in diverse signaling pathways [1,2]. While localized in the cytosol, the AhR is held stably in an open ligand-binding conformation by its association with a complex of co-chaperone proteins, including two molecules of hsp90 (heat shock protein 90kDa), XAP2 (HBV X-associated protein 2, also called the AhR-interacting protein (AIP)), and p23 (phosphoprotein 23kDa) [3,4,5,6]. Binding of ligand within the cavity of the AhR ligand binding domain (LBD) triggers conformational changes in the AhR structure that exposes a nuclear localization sequence and stimulates nuclear translocation of the liganded AhR protein complex. Within the nucleus, the dimerization of AhR with the Ah receptor nuclear translocator (ARNT) protein causes dissociation of the AhR-associated proteins and conversion of the AhR into its high-affinity DNA binding form [7]. This process is referred to as AhR transformation, and it effectively enables the ligand: AhR: ARNT complex to bind to its specific DNA binding site, the dioxin responsive element (DRE), adjacent to target genes and mediate AhR-dependent gene expression [8,9]. Recent studies have also reported that the AhR can heterodimerize with other nuclear proteins (KLF6 and RelB) and stimulate gene expression via distinctly different DNA binding sites [10,11,12].

Similar to the ligand-activated pregnane X receptor (PXR), a member of the steroid, thyroid and retinoic acid receptor superfamily, the AhR is promiscuous in its propensity to bind structurally diverse compounds, and significant differences in ligand binding specificity and ligand-specific AhR functionality have been reported across species [13]. The promiscuity reported for PXR ligands has been shown to result from the ability of these structurally diverse ligands to differentially bind to residues within the PXR ligand binding cavity [14,15]. By analogy, differences in the binding of ligands within the AhR LBD could also account for the observed structural diversity of AhR ligands, and this possibility is supported by several recent reports [16,17,18]. Examination of polymorphisms and mutations within the murine AhR (mAhR) LBD has identified several residues that contribute to some of the observed differences in affinity and selectivity of AhR ligands [17,18,19]. For example, while the ability of TCDD and related high-affinity halogenated and polycyclic aromatic hydrocarbon (HAHs and PAHs, respectively) ligands to activate the mAhR is significantly reduced by mutation of histidine 285 to tyrosine (H285Y), the structurally divergent ligand YH439 still activates the AhR [18]. Soshilov and Denison [17] not only identified a distinct region of the mouse AhR LBD (inclusive of amino acids H285, F289, F318, and H320) that was responsible for ligand-specific activation of the AhR, but mutations of isoleucine 319 (I319) into various other amino acids revealed significant diversity in AhR ligand selectivity. These results are consistent with differential interactions of structurally diverse agonist ligands with residues within the AhR ligand binding cavity. Interestingly, mutation of phenylalanine 318 (F318) revealed a role for this residue in differentiating between agonist and antagonist modes of action of a chemical [17]. Identification of antagonists that are species-specific [20,21] and/or ligand-selective [22] is also consistent with differential binding of chemicals within the AhR LBD, which can allow for ligand-selective activation or inhibition of the AhR [10].

In-depth analyses of the human AhR (hAhR) have demonstrated that it exhibits distinct differences in ligand-binding affinities, transformation efficiency, gene expression, and downstream biological and toxic effects when compared to rodent AhRs [23,24]. Although the hAhR was once considered an impaired AhR isoform due to reduced ligand binding and differential gene induction following activation by classical AhR ligands (2,3,7,8-tetrachlorodibenoz-p-dioxin (TCDD, dioxin) and polycyclic aromatic hydrocarbons), detailed molecular analysis revealed that a single amino acid difference (position 375 in the mAhR) between the “high-affinity” AhR (alanine in the AhR^b^ allele) and “low-affinity” AhR (valine in the mAhR^d^ allele and present in the hAhR) was responsible [25,26]. This was confirmed when mutation of A375 in the high-affinity mouse AhR to valine (A375V) resulted in a ~10-fold lower affinity and activation potency of the mAhR by TCDD [27,28], making the mAhR more similar to that of the hAhR. Significant species differences in AhR ligand-binding specificity and potency have also been reported more recently [23,24,26,29,30]. For example, while the AhR agonist indirubin (IR) has been shown to have a 10-fold greater potency as an inducer of AhR-dependent gene expression than TCDD in human hepatoma cells, it is ~10-fold less potent than TCDD in mouse hepatoma cells [23]. Additionally, IR was shown to be a more efficacious activator than TCDD of the hAhR in vitro and in cells in culture. For ligand-specific differences in hAhR response to manifest in a single cell type, significant differences in the binding interactions between ligands and amino acids within the hAhR LBD must occur, and this must translate into alterations in hAhR and/or hAhR: hARNT structure/function that ultimately lead to distinct ligand-dependent differences in AhR response. The documented ability of the AhR antagonist CH223191 to inhibit TCDD- but not IR-dependent gene expression in rat hepatoma cells suggests that these two ligands differentially interact with the AhR LBD [22]. However, the specific interactions and mechanisms responsible for, or contributing to, the enhanced potency and response of the hAhR to IR remains to be elucidated. We hypothesize that the enhanced potency of IR as an agonist of the hAhR, compared to the mAhR, results from species-specific differences in the interaction of IR with residues within the human and mouse AhR LBDs, but the specific interactions remain to be identified. Here we describe the results of studies using a combination of site-directed mutagenesis and AhR functional analysis to examine the mechanism(s) responsible for differences in the ability of IR to bind to and activate the hAhR and mAhR.

## 2. Results

### 2.1. Comparison of the Relative Potency and Efficacy of AhR Agonists in Human and Mouse Cells

**Figure 1:**
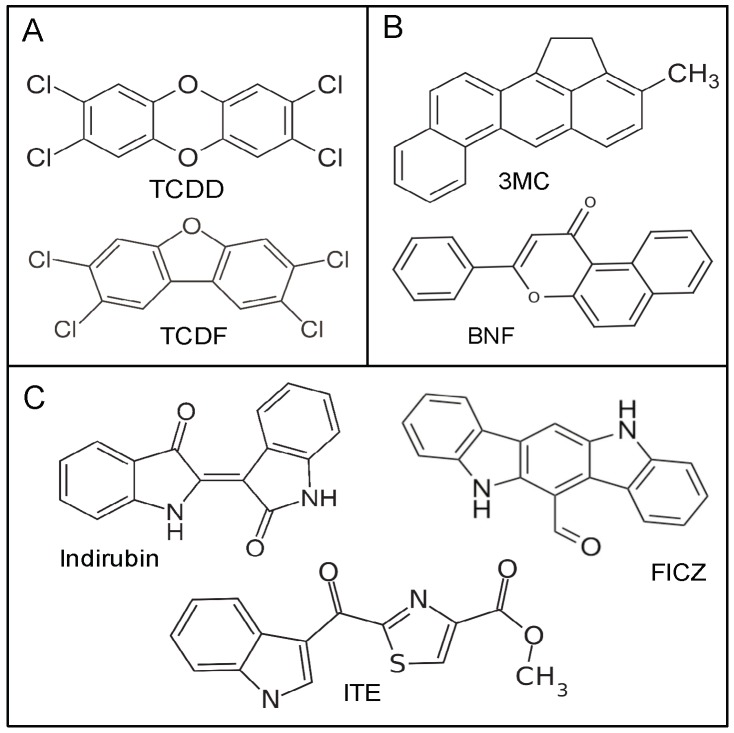
Structures of the AhR agonists examined for species specificity. (**A**) Halogenated aromatic hydrocarbons (HAHs); (**B**) Polycyclic aromatic hydrocarbons (PAHs) and PAH-like; (**C**) indole-containing compounds.

**Figure 2:**
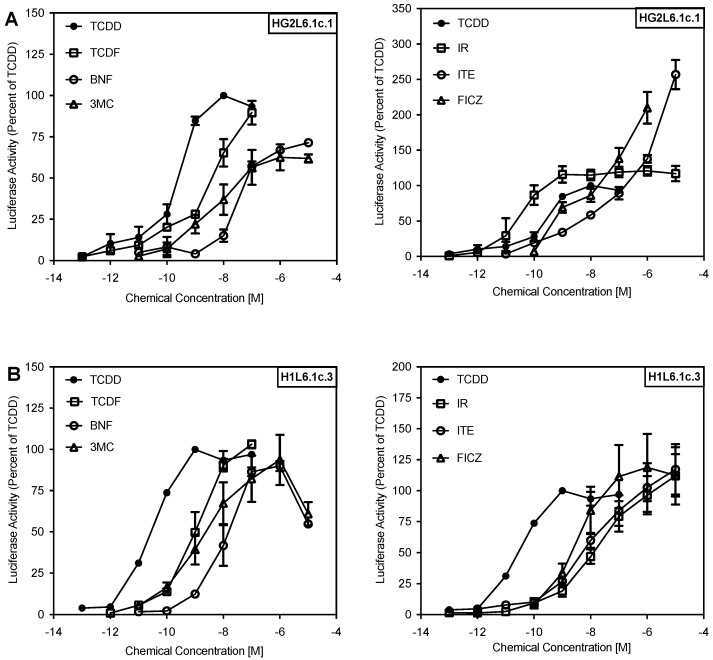
IR preferentially induces reporter gene transcription in HG26.1c1 cells. AhR-dependent gene transcription was determined in (**A**) human HG2L6.1c.1 and (**B**) mouse H1L6.1c.3 hepatoma cell lines that contain a stably transfected AhR-responsive luciferase reporter gene. Cells were incubated with DMSO (1%, *v*/*v*), TCDD (0.1–100 nM), TCDF (0.1–100 nM), BNF (0.001–10 µM), 3MC (0.001–10 µM), IR (0.001–10 µM), ITE (0.001–10 µM), and FICZ (0.001–10 µM) for 4 h. Luciferase activity (Relative Light Units (RLUs)) was normalized to the maximal induction observed with TCDD in each cell line. Values represent the mean ± SD of nine independent analyses.

IR has been reported to preferentially stimulate the hAhR [23,29,31,32,33]; however, whether this phenomenon is specific for IR or whether other AhR ligands show similar species differences remains to be determined. In initial experiments, we examined the ability of several structurally diverse chemicals (Figure 1) to activate AhR-dependent gene expression in mouse and human cells. TCDD, TCDF, BNF and 3-MC induced luciferase reporter gene expression in human HG2L6.1c1 cells in a concentration-dependent manner (Figure 2), although maximal induction was not observed with BNF or 3-MC in these cells. Not only do these results reveal a similar rank order potency for these compounds in both mouse and human cells, but the relative inducing potency of each compound was ~10-fold less in the human HG2L6.1c1 cells (Supplemental Table S1). Interestingly, distinct species difference in the rank order potency of reporter gene induction were observed by IR, ITE and FICZ. In mouse H1L6.1c3 cells, these compounds stimulated reporter gene induction to maximal levels comparable to that with TCDD and they were between 50–250-fold less potent than TCDD. In contrast, in human HG2L6.1c1 cells, IR was ~7-fold more potent than TCDD, FICZ was equipotent to TCDD and ITE was ~100-fold less potent than TCDD (Figure 2; Supplemental Table S1). Additionally, these three compounds induced reporter gene expression in human cells to a level significantly greater than that obtained with TCDD alone; a phenomenon known as ‘superinduction’ of AhR-dependent gene expression relative to TCDD. While the exact mechanism(s) responsible for the observed superinduction response is not known, we have previously described several possible mechanisms that may contribute to synergistic increases in AhR-dependent gene expression [34]. These results are not only consistent with differences in the ability of IR, ITE and FICZ to bind to the human and mouse AhRs, but they demonstrate that of the tested compounds, IR exhibited the greatest species-specific differences in response. Accordingly, further studies focused on investigation of species differences in IR-dependent activation of the hAhR and mAhR.

### 2.2. The Human AhR Ligand Binding Domain Is Essential for Ligand-Selective Activation by IR

**Figure 3:**
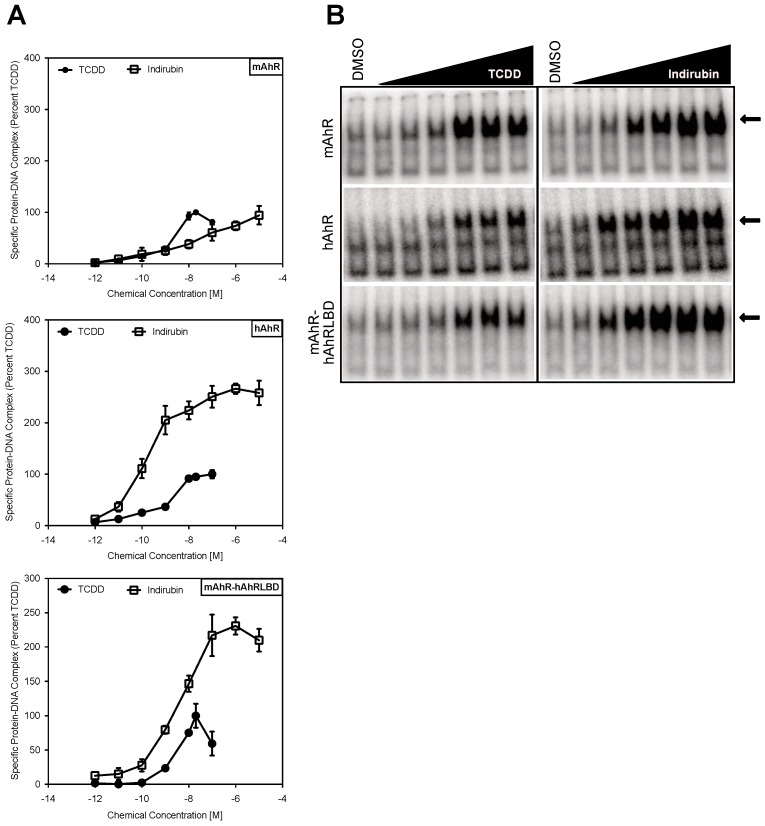
The human AhR ligand binding domain plays a major role in ligand-selective activation by IR. In vitro synthesized wild-type or chimeric AhRs and ARNT were incubated in the presence of solvent control DMSO (1%, *v*/*v*) or AhR agonists TCDD (0.001–100 nM) or IR (0.001–10,000 nM) for 2 h and analyzed by gel retardation assay. (**A**) The amount of inducible protein-DNA complex at each chemical concentration was quantitated and values normalized to the amount of complex formed with a maximal activating concentration of TCDD (20 nM). (**B**) Representative gels are shown. Values represent the mean ± SD of nine individual replicate analyses. The arrows indicate the ligand-induced protein-DNA complexes.

**Figure 4:**
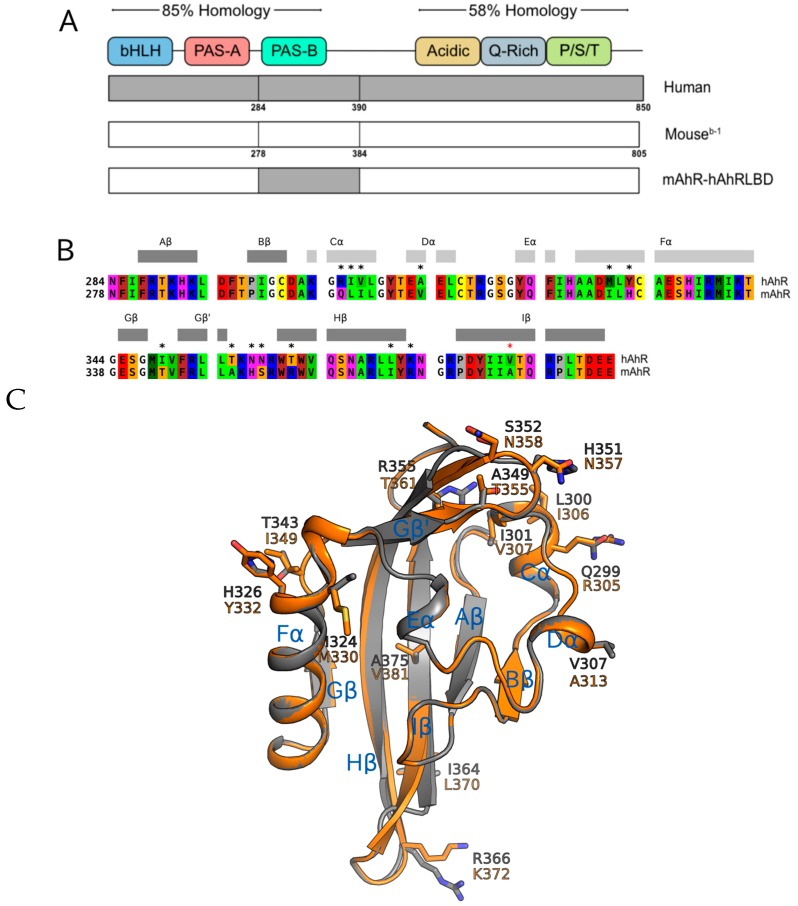
Comparison of the AhR PASB ligand binding domain of the human and mouse AhR. (**A**) Structural domains of the hAhR (850 aa), mAhR (805 aa) and chimeric mAhR-hAhRLBD (811 aa) proteins; (**B**) Sequence alignment of the mAhR and hAhR PASB LBDs with non-consensus residues indicated by asterisks (red asterisk for the internal different residue). Coloring scheme for residues: red, acidic; blue, basic; purple, polar; yellow, Cys; brown, aromatic; green, hydrophobic; orange, Ser, Thr; gray, Pro; white, Gly. Secondary structure elements according to DSSP for the modeled AhR LBDs are reported above the sequences (helices: light gray; β-strands: dark gray) and labeled with the PAS structure nomenclature; (**C**) Cartoon representation of the AhR LBD homology models (structural superposition of: mAhR, in gray, hAhR, in orange). Nonconserved residues are shown as sticks. Secondary structure elements are labeled with the PAS structure nomenclature.

Ligand-selective and species-specific differences in AhR activation suggest that the diversity in response may result from differential binding of structurally diverse ligands within the binding cavity of the AhR LBD [1,17,18,22,28]. To examine this, we first compared the ability of IR and TCDD to stimulate in vitro transformation and DNA binding of the mAhR and hAhR by gel retardation analysis. These results not only revealed that IR can stimulate transformation and DNA binding of both in vitro synthesized mouse and human AhRs (Figure 3), but that IR was a more potent and efficacious activator than TCDD of the hAhR, similar to the gene expression results (Figure 2). To confirm that the increased responsiveness of the hAhR was due to its LBD and not to other regions of the AhR, we examined the ability of TCDD and IR to stimulate DNA binding of a chimeric mAhR (mAhR-hAhRLBD) in which the mAhR LBD has been replaced with the corresponding region of the hAhR LBD (Figure 4A). These analyses revealed that the hAhR LBD was essential for the significantly enhanced DNA binding signal observed with IR (Figure 3). While the relative potency of TCDD was similar between the hAhR and mAhR-hAhRLBD chimera, the lower degree of TCDD-stimulated DNA binding compared to IR suggests that differences in the efficacy of each compound as AhR activators may account for the diminished reporter gene transcription reported in human cells [23]. Additionally, although the relative potency of IR in the chimeric AhR was not as dramatically different from that of TCDD, as observed with the hAhR, it was still more potent. These results suggest that the hAhR LBD plays an essential role in ligand-selective activation by IR. However, since the mAhR-hAhRLBD did not completely recapitulate the potency difference in IR activation of the hAhR, it is conceivable that additional regions of the hAhR may play a role and/or that protein-protein interactions of the hAhR with ARNT or other partners may contribute to enhancing IR-dependent hAhR transformation efficiency and DNA binding.

### 2.3. Point Mutations in the mAhR LBD Produce a hAhR-Like Response with IR

Given the highly conserved nature of the AhR across species and the role of the hAhR LBD in ligand-selective activation by IR, it is likely that amino acid differences within the hAhR are responsible for the enhanced activation by IR. Sequence analysis of the LBDs of the mouse and human AhRs identified 13 nonconserved residues (Figure 4B,C). Among these, only the mAhR A375 (valine in the hAhR) has the side-chain directly pointing into the AhR binding cavity previously determined by homology modeling (Figure 4C) and confirmed by mutational analysis [27,28]. To determine which of the nonconserved residues play a role in the enhanced response of the hAhR to IR, a series of mutations within the mAhR LBD were generated, wherein residues in the mAhR were mutated to the corresponding hAhR LBD residues. The relative ability of increasing concentrations of IR and TCDD to stimulate transformation and DNA binding of the mutant mAhRs was determined by gel retardation analysis.

**Figure 5:**
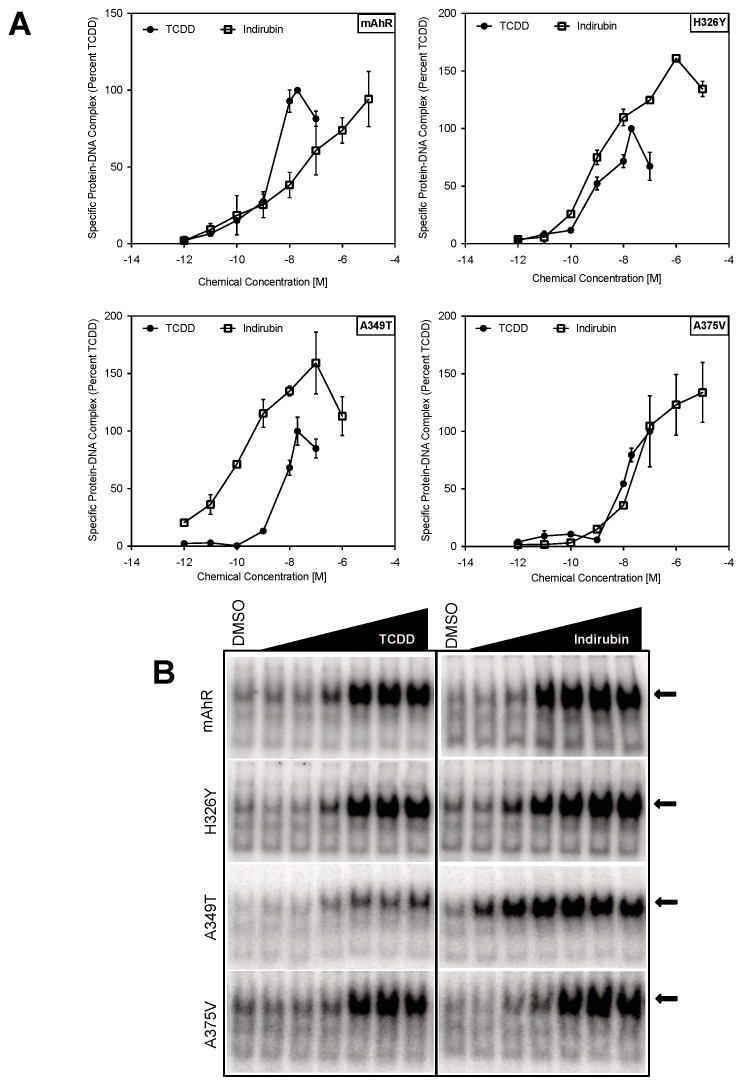
Enhanced signaling of the hAhR by IR is associated with selected residues within the hAhR PASB LBD. In vitro synthesized mutant mAhRs and wild-type mARNT were incubated in the presence of DMSO (1%, *v*/*v*) or AhR agonists in DMSO (TCDD (0.001–100 nM) or IR (0.001–10,000 nM) for 2 h, followed by analysis of AhR DNA binding by gel retardation analysis. (**A**) The amount of inducible protein-DNA complex at each TCDD or IR concentration was quantitated, and values normalized to the amount of complex formed with a maximal activating concentration of TCDD (20 nM). (**B**) Representative gels are shown. Values represent the mean ± SD of nine individual replicate analyses. The arrows indicate the ligand-induced protein-DNA complexes.

These experiments identified two mAhR to hAhR mutations (H326Y and A349T) that enhanced the efficacy of IR-dependent DNA binding of the mAhR by IR, and one mutation (A375V) that increased the relative potency, but not efficacy, of IR, such that IR was now equipotent to that of TCDD (Figure 5). The remaining ten mutations did not significantly alter IR activation from that of wild-type mAhR (Supplemental Figure S1. Mutation of histidine 326 to tyrosine (H326Y) and alanine 349 to threonine (A349T) enhanced the ability (efficacy) of IR to stimulate transformation of the mAhR into its high-affinity DNA binding form and increased the relative potency of IR for the mAhR to greater than that of TCDD. The A349T mutation also resulted in the greatest increase in the relative potency of IR as compared to TCDD (Figure 5) and this appeared to result primarily from a significant decrease in the ability of TCDD to stimulate AhR DNA binding. Interestingly, the A349T mAhR mutant almost entirely recapitulated the enhanced response of hAhR to IR (Figure 3).

**Figure 6:**
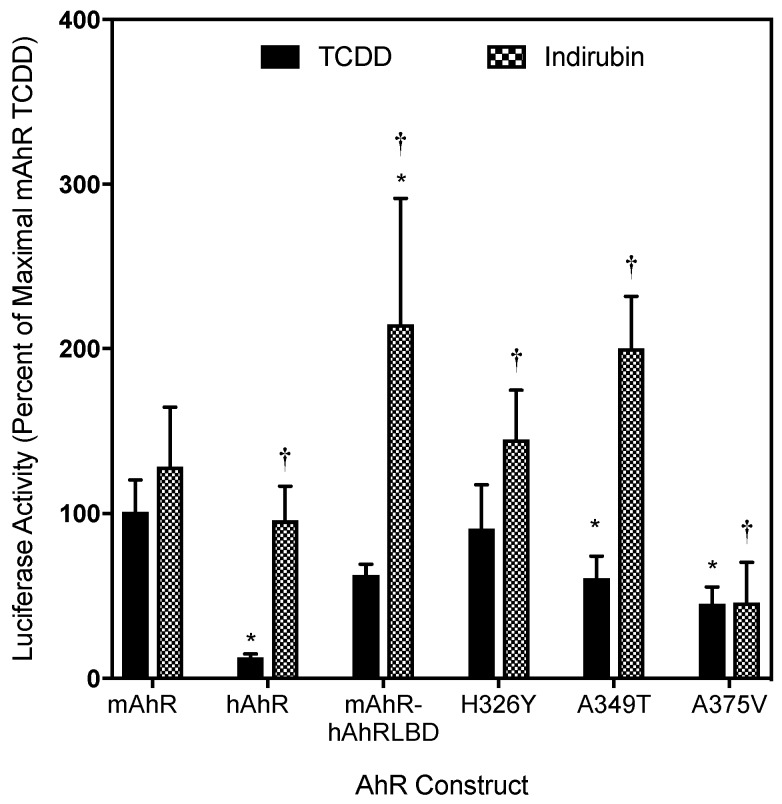
mAhR point mutations alter AhR-dependent reporter gene transcription following TCDD and IR treatment. COS-1 cells transiently transfected with wild-type or mutant mAhR and DRE-containing reporter pGudLuc6.1 were treated with solvent control DMSO (0.1%, *v*/*v*), TCDD (10 nM), or IR (1 µM) for 18–20 h. Cells were lysed, and lysates were analyzed for firefly luciferase activity. Asterisks indicate the values that are significantly different from the mAhR TCDD or IR, as indicated by Two-Way ANOVA with *p* < 0.05 and (†) indicate IR values are significantly different from TCDD within the given construct by Student’s *t*-test *p* < 0.05. Luciferase activity (relative light units; RLU) was measured and corrected for background (DMSO) and normalized to mAhR TCDD levels. Values represent the mean ± SD of nine individual replicate analyses.

Consistent with these observations are the induction results obtained in COS-1 cells cotransfected with wild-type or mutant (H326Y, A349T and A375V) mAhR expression vector and the AhR-responsive luciferase reporter plasmid pGudLuc6.1. While TCDD was comparable to IR as an inducer of luciferase activity in COS-1 cells cotransfected with wild-type mAhR, IR was a significantly more efficacious inducer than TCDD in cells cotransfected with the hAhR, the mAhR-hAhRLBD and the mutant A349T mAhR (Figure 6). Similar to the DNA binding results, while the maximal induction observed with TCDD in cells cotransfected with the A349T mutant mAhR was significantly repressed compared to wild-type mAhR, the maximal induction response observed with IR was significantly increased above that of wild-type mAhR and of that of TCDD. This decreased response likely results from the reduced ability of TCDD to stimulate transformation and/or DNA binding of mAhR containing this substitution (Figure 5). The relative enhancement of reporter gene induction by indirubin, compared to TCDD, in these experiments was somewhat lower than its relative response in the earlier studies (Figure 3). However, this was not surprising and likely results from a combination of factors. First, the experiments in Figure 3 not only utilized stably transfected human hepatoma cells containing a wild-type hAhR (with numerous amino acid differences from the mAhR), but cells were incubated with IR for only 4 h before measurement of luciferase activity. In contrast, the experiments presented in Figure 6 utilized COS-1 cells transiently transfected with a mAhR that contained a single amino acid substitution in the LBD based on the hAhR and induction of luciferase activity was examined after 18–20 h, which allowed the cells more time to metabolically degrade the IR. The dramatic difference in the induction response of TCDD and IR with the A349T mAhR is not only consistent with their respective abilities to stimulate AhR transformation and DNA binding, but it suggests that there are significant differences in how these specific ligands interact with residues within the AhR ligand binding cavity and/or how their binding may translate into differences in AhR transformation/DNA binding.

### 2.4. Ligand Binding Analysis Reveals That the A349T Mutation Differentially Affects TCDD and IR

**Figure 7:**
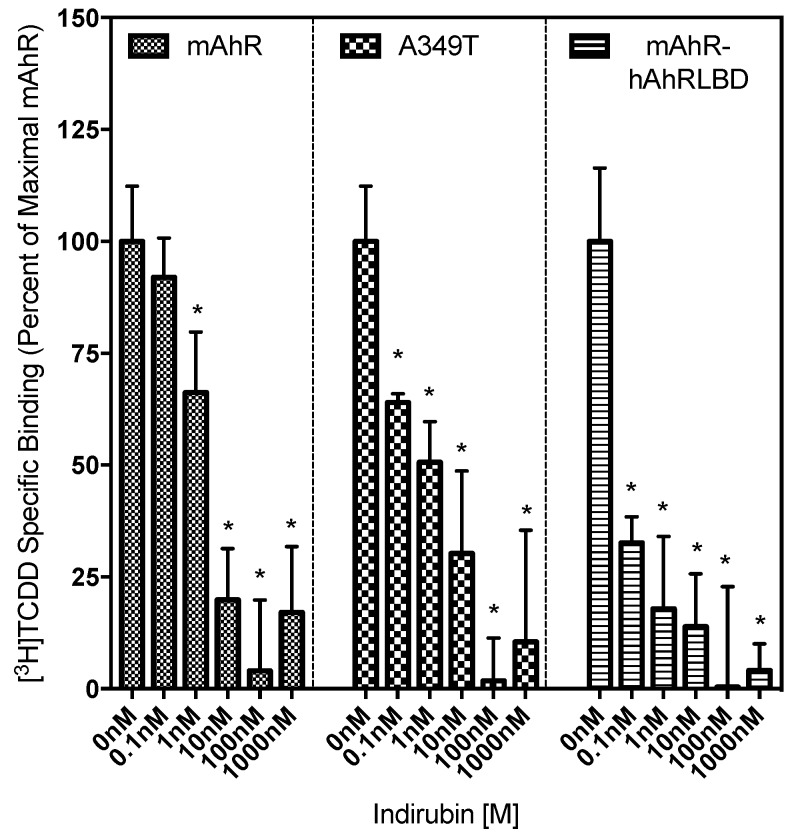
A349T mutation has no significant effect on the relative affinity of IR for mAhR. In vitro synthesized mAhR, mutant AhR, or mAhR-hAhRLBD chimeric protein was incubated in the presence of 2 nM [^3^H]TCDD and indicated concentrations of IR for 30 min and [^3^H]TCDD bound to the protein fraction was measured by the hydroxyapatite assay. The unprogrammed TNT lysate was used as a nonspecific binding control, and specific binding was calculated as a difference between the total and nonspecific reactions. Values represent the mean ± SD of nine individual replicate analyses. The asterisks indicate the values that are significantly different from the no-competitor reaction, as indicated by the Two-Way ANOVA with *p* < 0.05. The relative affinity of IR for the mAhR, A439T and mAhR-hAhRLBD was 17.67 ± 1.66, 4.61 ± 1.87 and 0.82 ± 0.28 nM, respectively, as determined using nonlinear regression (three-parameter) analysis of the competitive binding results.

While the above results demonstrate that the insertion of an A349T mutation in the mAhR LBD significantly reduced the ability of TCDD to stimulate AhR transformation/DNA binding and/or AhR-dependent gene expression, this mutation enhanced the activity of IR. However, whether these effects result from an alteration in the ability of these chemicals to bind to the AhR is unknown. Accordingly, competitive [^3^H]TCDD ligand binding analysis was carried out to determine whether the A349T mutation alters the relative ability of [^3^H]TCDD or IR to bind to the mAhR. In these experimental conditions, although ~10% more [^3^H]TCDD bound to the in vitro synthesized wild-type mAhR than to the mAhR-hAhRLBD chimera, [^3^H]TCDD binding to the A349T mutant mAhR was not significantly different from that of the wild-type mAhR, indicating that this hAhR-specific mutation had no significant effect on overall [^3^H]TCDD specific binding. The relative ability/affinity of IR to bind to each of these AhRs was then determined by concentration-dependent [^3^H]TCDD competitive binding analysis (Figure 7). These results not only revealed that IR could bind to the mAhR-hAhRLBD chimera with an apparent affinity greater than that for the mAhR (IC_50_ values of 0.82 ± 0.28 nM and 17.67 ±1.66 nM, respectively), but insertion of the A349T mutation in the mAhR had no significant effect on the affinity of binding of IR (IC_50_ value of 4.61 ± 1.87 nM) (Supplemental Table S3). Interestingly, although the A349T substitution had no significant effect on the binding of TCDD or IR to the mAhR, a significant reduction in the ability of TCDD and an increase in the ability of IR to stimulate AhR transformation/DNA binding and AhR-dependent gene expression was observed. While A349 does not appear to be involved in AhR ligand binding, or at least the affinity of binding, the increased DNA binding response suggests that it plays a role in ligand-stimulated AhR transformation/DNA binding.

### 2.5. Molecular Docking Predicts Differences in TCDD and IR Binding within the mAhR and hAhR LBDs

**Figure 8:**
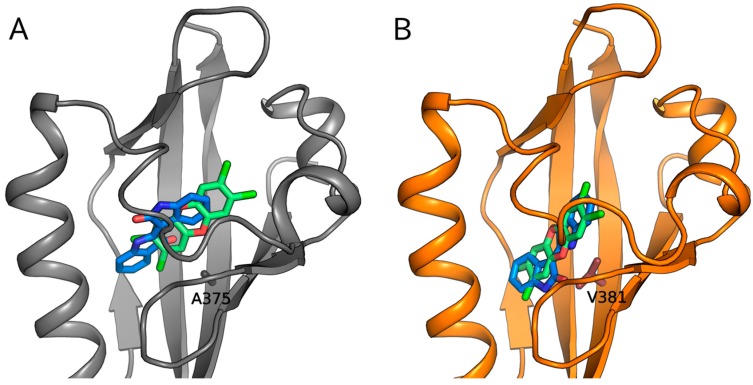
Binding geometries of TCDD (light green) and indirubin (marine blue) predicted by molecular docking to the AhR PASB LBD homology models. (**A**) mAhR in grey and (**B**) hAhR in orange. The nonconserved internal residues A375/V381 are shown as sticks.

To analyze the hypothesis that IR interacts with residues within the AhR ligand binding cavity in a manner distinctly different from that of TCDD, the binding geometries of these ligands in the mAhR and hAhR LBDs were predicted by molecular modeling methods. The LBD structures previously generated by homology modeling [35] were used for molecular docking, and the three forms of IR were submitted to docking simulations (see Materials and Methods section). The most energy-favored complex was obtained with the IR trans stereoisomer for both mAhR and hAhR. The docking poses of TCDD and IR in the mAhR LBD (Figure 8A) confirm that IR binds within the cavity with different arrangement and interactions compared to TCDD. While TCDD occupies the central part of the cavity and the two chlorine atoms reach the most internal hydrophobic region [35], IR binds nearer to the entrance of the cavity. This causes the loss of some stabilizing interactions that are characteristic of TCDD binding with residues in the most buried and hydrophobic region within the cavity but, at the same time, it allows a gain of stabilizing contributions with residues near to the cavity entrance (Fα and Gβ). While the naturally occurring mutation of A375 (mAhR) to V381 (hAhR) causes the displacement of the TCDD binding pose toward the entrance of the hAhR cavity (35), it slightly affects the IR pose by maintaining the same placement observed in the mAhR cavity with a modified orientation of the molecular plane (Figure 8B). Therefore, the IR molecule contacts some of the residues predicted for the mAhR pose at the cavity entrance as well as some unique ones. Despite the similar placement of TCDD and IR at the entrance of the hAhR binding cavity (Figure 8B), the distinct chemical characteristics of the two ligands generate different stabilizing interactions with the internal residues.

## 3. Discussion

It is well established that the AhR can bind and be activated by a wide range of structurally diverse compounds and that significant species differences exist in ligand specificity [1,2,36,37]. The emergence of endogenous AhR ligands that are both structurally diverse and stimulate physiological signaling pathways, highlights the importance of understanding the mechanisms by which these compounds selectively activate the human AhR relative to classical rodent models for the purpose of improving risk management strategies and development of AhR pathway-specific therapeutics. Endogenous AhR activator, indirubin, has been detected at concentrations of 0.2 nM in human urine and shown to modulate AhR-dependent microbiome homeostasis and inflammatory signaling pathways [24,29,30,31]. Potent induction of hAhR-dependent gene transcription in transgenic mice, transfected mouse hepatocytes, and human hepatoma cells indicates that IR preferentially activates the human AhR [23]; however, the mechanism of ligand-selective activation and whether structural divergence within the ligand binding domain plays a role in regulation of hAhR functionality had not been thoroughly investigated.

By analogy with mechanisms that have been reported for ligand-dependent gene expression by steroid hormone receptors, ligand- and species-specific differences in the activation of the AhR could alter AhR and ARNT protein structure and thus influence recruitment/interaction with coactivators, transformation efficiency, protein-protein interactions, gene expression, and downstream signaling events. The ability of IR to enhance transformation and DNA binding efficiency in the hAhR and mAhR-hAhRLBD chimeric construct in vitro, relative to the mAhR, confirmed the significance of the hAhR LBD on conferring IR-dependent ligand-selective activation of the AhR. Insertion of the hAhR LBD into the mAhR did not completely recapitulate the hAhR phenotype, suggesting that the full enhanced response to IR may require additional human-specific AhR residues, protein-protein interactions and/or protein conformational changes that only occur within the full length hAhR and not in the mAhR-hAhRLBD chimera. In fact, it has been previously shown that the mAhR LBD coordinates with the C-terminal end to effectively transform the receptor and enable enhancement/repression of target gene transcription [24]. Likewise, the effects of species-specific LXXLL coactivators, interdomain influence, or crosstalk with other nuclear proteins could modulate AhR-dependent responsiveness to a given ligand across species [38]. The premise that the structure and functional activity of the AhR is dependent on the specific ligand bound within the cavity is supported by gene expression studies, where diverse AhR ligands produce distinctly different magnitudes of induction of the same gene at equipotent concentrations and also induce a ligand-specific set of AhR-dependent gene products [1,39]. While the majority of the known selective activators of the hAhR (i.e., indoxyl sulfate, indole, tryptamine, and 2AI) are indole-containing compounds [30], other selective AhR activators have also been identified. Cinnabarinic acid (CA), which is structurally similar to TCDD and does not contain an indole group, demonstrates clear ligand-selective AhR-dependent activation of stannocalcin 2 (Stc2) gene expression [39,40]. Chromatin immunoprecipitation analysis of the Stc2 promoter region identified several DREs that were selectively bound by CA-activated AhR and induced CA- and AhR-dependent induction of Stc2, while classical ligands (TCDD, β-naphthoflavone, 3-methylcholanthrene) failed to stimulate AhR binding and Stc2 gene expression [39]. The selective activation of the AhR by these and other structurally diverse AhR ligands (agonists and antagonists) suggests alternate binding within the AhR ligand binding domain, which could potentially stimulate distinct AhR conformational changes and downstream biological effects [18,21].

Site-directed mutagenesis and functional analysis of a series of mutant mAhRs that contained a single amino acid substitution in its LBD that was swapped for the corresponding residue in the hAhR LBD allowed identification of 3 individual mutations inserted into the mAhR LBD (H326Y, A349T and A375V) that significantly increased the potency of IR relative to that of TCDD (Figure 5 and Supplemental Table S2); the remaining individual mutations had little or no significant effect on the relative potency of TCDD and IR. While one mutation (A349T) significantly increased the relative potency of IR from ~6-fold less potent than TCDD to ~50-fold more potent than TCDD, the remaining two mutations (H326Y and A375V) increased the relative potency of IR such that it became equipotent to TCDD. It should be noted that while the H326Y substitution actually had a greater effect on the relative potency of IR (compared to TCDD) than the A375V mutation, because the latter mutation also resulted in a significant decrease the relative potency of TCDD for the mAhR. The reduction in the affinity of the AhR for TCDD with this specific mutation was previously reported [27,28]. Overall, these results, combined with previous site-directed mutagenesis analysis [17] demonstrate that significant differences in binding do exist between TCDD and IR and suggest that the selectivity and responsiveness to IR may depend on multiple amino acids within the LBD.

So how might these specific residues within the hAhR contribute to the specificity and increased potency of IR compared to that of TCDD? Based on our homology models of the mAhR LBD [35], two of these residues (H326 (Y332 in the hAhR) and A349 (T355 in the hAhR)) are in distinctly different locations within the LBD. H326 is located in the Fα helix or long helical connector of the PASB (Figure 4C), which in other PAS proteins plays an important signaling role and can be displaced from the β-sheet in different ligand-dependent ways to form a cavity suitable for a variety of ligands or cofactors [41,42]. Since the side chain of H326/Y332 points out of the binding cavity and toward the G-strand of the AhR β-sheet, mutation of this residue would not be expected to affect AhR ligand binding; however it could alter AhR functionality by altering ligand-selective transformation and DNA binding and thereby affecting the interaction of AhR with its partner proteins (e.g., hsp90 and/or ARNT). This hypothesis is supported by our recent models of the AhR:ARNT dimer in which the helical connector of the mAhR PASB is involved in the dimerization interface [43,44]. The homology model of the AhR LBD indicates that the A349 residue is located in a loop connecting the G and H strands of the β-sheet and similar to H326, its side chain does not point into the AhR ligand binding cavity (Figure 4C). However, given the effect of its substitution on IR-dependent DNA binding, this residue must play a role in the enhanced ability of IR, compared to TCDD, to stimulate transformation/dimerization of the AhR with ARNT and conversion of the AhR into its DNA binding form. However, since our recent modeling of AhR: ARNT dimerization [43,44] indicates that this residue does not appear to be involved in the dimerization interface with ARNT, we infer that it plays a more significant role in interactions with other partners or with different regions of the AhR and ARNT proteins that enhance the ability of IR to stimulate AhR transformation/DNA binding. The presence of valine at position 381 in the hAhR LBD has been documented to significantly reduce the binding affinity of TCDD due to steric hindrance by its side chain pointing directly in the center of the modeled LBD cavity, likely disrupting the interaction of TCDD with a closely associated histidine residue [27,28]. Mutation of alanine in this position in the mAhR to valine decreases the binding affinity of TCDD [27,28]. Interestingly, while it was reported that mutation of this residue in the hAhR (V381A) or mAhR (A375V) had no effect on the binding of IR to the AhR [23], the gel retardation analysis results presented here (Figure 5 and Supplemental Table S2) revealed that a A375V mutation in the mAhR increased the relative potency of IR in stimulating mAhR transformation and DNA binding ~5-fold. Together, these results are consistent with significant differences induced by in the binding of IR and TCDD within the cavities of these AhRs, with IR binding affinity apparently unaffected by mutation of this residue. 

While the exact mechanism(s) by which TCDD and IR bind to and activate the AhR remain to be elucidated, the results presented here demonstrate that the differential interactions of these ligands with residues within the LBD of the hAhR are responsible for the enhanced signaling potency of IR. One avenue in which to gain additional insights into similarities and differences in the interaction of TCDD and IR with residues inside the binding cavities of the hAhR and mAhR is by performing molecular docking of these ligands into the homology models of their LBDs [45]. Docking analysis of TCDD within both the AhR LBDs has been previously reported by our laboratory [35], and the docking simulations of IR binding presented here have allowed prediction of the binding geometries and direct comparison with those of TCDD. Despite the fact that IR maintains the characteristic planarity of TCDD and other dioxin-like AhR ligands, it shows different molecular dimensions and shape, higher polarity, and has functional groups that may form hydrogen bonds. As a consequence, our docking analysis revealed binding geometries of IR in the mAhR LBD distinctly different from that of TCDD (Figure 8A). While TCDD showed stabilizing interactions with residues both in the middle and in the inner part of the cavity [35], the IR binding site was predicted to be closer to the entrance of the cavity and this resulted in different stabilizing interactions of IR with internal residues. Interestingly, docking to the hAhR LBD revealed that the V381 residue, known to adversely affect the binding of TCDD [27,28], perturbs both the TCDD and IR binding poses causing a similar placement of the two ligands within the cavity (Figure 8B). TCDD is displaced toward the cavity entrance and loses many of the stabilizing interactions that occur in the mAhR cavity. In contrast, the V381 residue does not hinder the binding of IR, which occupies a region near to the entrance of the cavity like that in the mAhR, but only induces a rotation of the molecular plane. Taken together, these findings suggest that alanine or valine similarly affect the binding affinity of IR to the AhR in agreement with experimental evidence [23] and suggest that IR may have a higher relative binding affinity to the hAhR compared to TCDD. This prediction is consistent with the [^3^H]TCDD competitive binding results that suggest higher binding affinity of IR to the hAhR LBD. The differential binding poses of IR and TCDD within the cavity also likely contribute, along with other sequence and structural differences in the AhRs, to the subsequent transformation events responsible for the enhanced potency and efficacy of IR.

Site-directed mutagenesis and structure-function analysis, including those based on homology models, have aided in providing clear evidence for differential binding of structurally diverse ligands within the mouse and human AhR ligand binding cavity. Although part of the AhR: ARNT dimer structure (bHLH-PASA) was experimentally determined recently [46,47], the lack of experimental 3D structures of both the AhR PASB LBD and the full length AhR: ARNT dimer including this domain still hampers a complete mechanistic understanding of binding by structurally diverse ligands and how ligand binding activates the AhR. Targeting of the AhR and/or AhR signaling pathway holds significant promise for development of therapeutic pharmaceuticals for treatment of a variety of human diseases. Thus, future studies identifying and characterizing the residues responsible for ligand-dependent AhR transformation, ARNT-dimerization, DNA binding and transcriptional activation will be crucial for increasing our understanding of the basic molecular mechanisms underlying the functional activity of the AhR and its diversity in biological and toxicological responses.

## 4. Materials and Methods

### 4.1. Chemicals

TCDD was obtained from Dr. Stephen Safe (Texas A&M University), [^3^H]TCDD (14.3 Ci/mmol) was obtained from ChemSyn Laboratories (Lenexa, KS, USA), and 2,3,7,8-tetrachlorodibenzofuran (TCDF) was from Accustandard (New Haven, CT, USA). [^32^P]-ATP (~6000 Ci/mmol) was from Perkin Elmer Life & Analytical Sciences. The structures of the specific AhR ligands used in these studies are shown in Figure 1. 3-Methylcholanthrene (3MC), β-naphthoflavone (βNF) and dimethyl sulfoxide (DMSO) were from Sigma-Aldrich (St. Louis, MO, USA), 2-(1*H*-Indol-3-ylcarbonyl)-4-thiazolecarboxylic acid methyl ester (ITE) and 6-formylindolo[3,2-b]carbazole (FICZ) were from Tocris Bioscience (Minneapolis, MN, USA) and IR from AmplaChem (Carmel, IN, USA). All chemical stocks and dilutions were prepared in DMSO.

### 4.2. Plasmids

The mouse AhR and ARNT expression plasmids, mβAhR/pcDNA3 and mβArnt/pcDNA3, have been previously described [17]. The chimeric mouse/human mAhR/hAhR expression plasmid, mAhR-hAhRLBD, contains a full-length mAhR cDNA in which the mouse PASB LBD was replaced with the corresponding region of the hAhR LBD. Human AhR and ARNT were obtained from Christopher Bradfield (University of Wisconsin, Madison, WI, USA) and Oliver Hankinson (University of California, Los Angeles, CA, USA), respectively, and were inserted in pcDNA3β [26] to generate hβAhR/pcDNA3 and hβARNT/pcDNA3. Point mutations of mβAhR/pcDNA3 were designed using Agilent QuikChange Primer Design and Mus musculus aryl-hydrocarbon receptor transcript variant mRNA (NM_013464.4, nucleotides 367 to 2784). A list of mouse AhR mutagenic primers can be found in Table S4. Site-directed mutagenesis was carried out using the Agilent Technologies QuikChange Lightning Mutagenesis Kit and all constructs were verified by sequencing.

### 4.3. Hydroxyapatite [^3^H]TCDD Ligand Binding Assay

[^3^H]TCDD specific binding to wild-type mAhR and the chimeric mouse/human mAhR-hAhRLBD synthesized in vitro using the Promega TNT Quick coupled transcription/translation rabbit reticulocyte lysate kit (Madison, WI, USA) was carried out as previously described [48]. [^3^H]TCDD specific binding was determined by subtracting the amount of [^3^H]TCDD bound to unprogrammed lysate (nonspecific binding) from the total amount of [^3^H]TCDD binding to lysate containing in vitro expressed AhR. The amount of [^3^H]TCDD specific binding remaining in the presence of competitor chemical was expressed as a percent of the total [^3^H]TCDD specific binding.

### 4.4. AhR DNA Binding (Gel Retardation) Assay

Wild-type and mutant AhRs and ARNT were synthesized in vitro in the presence of unlabeled l-methionine, the resulting AhR and ARNT translation mixtures and MEDGK (25 mM MOPS (3-(N-morpholino) propanesulfonic acid; pH 7.5), 1 mM EDTA, 1 mM dithiothreitol, 10% (*v*/*v*) glycerol, 150 mM KCl) were mixed in a 1:1:8 (*v*/*v*/*v*) ratio and incubated with DMSO (1% final concentration) or the indicated concentration of TCDD or IR for the indicated periods of time at room temperature. An aliquot of each incubation was mixed with [^32^P]-labeled double-stranded oligonucleotide containing the AhR-ARNT DRE3 DNA binding site (a dioxin responsive element from the upstream region of the murine Cyp1a1 gene [9] and protein-DNA complexes resolved by gel retardation analysis as previously described [48,49]. Gels were visualized using a FLA9000 Fujifilm Imager and protein-DNA complexes quantitated with Fujifilm MultiGauge software (FujiFilm Corporation, Valhalla, NY, USA).

### 4.5. Reporter Gene Assays

Recombinant mouse (H1L6.1c3) and human (HG2L6.1c1) hepatoma cells containing a stably integrated AhR-responsive luciferase reporter gene plasmid (pGudLuc6.1) were plated (750,000 cells/well) in 96-well plates and incubated at 37 °C for 24 h prior to chemical treatment. Cells were incubated with DMSO (1% (*v*/*v*)) or the indicated concentration of test chemical in DMSO for 4 h, followed by visual inspection of the cells for toxicity, washing of the cells with phosphate-buffered saline (PBS), addition of 50 µL of passive lysis buffer (Promega) and lysis of cells for 20 min at room temperature with shaking [50]. Luciferase activity in each well was measured (integrating luminescence over 10 s with a 10 s delay) in an Orion microplate luminometer (Berthold Detection Systems, Bad Wildbad, Germany) following automatic injection of Promega stabilized luciferase reagent. Luciferase activity was corrected for background luciferase activity in DMSO-treated cells and values expressed as relative light units (RLU) or as a percent of the luciferase activity obtained with the maximally inducing concentration of TCDD.

### 4.6. Transient Transfection Assays

COS-1 cells were plated at a density of 75,000 cells/well and allowed to attach overnight in a 24-well plate. Cells were transiently transfected (per well) with the following amounts per well: 2 µL Lipofectamine 2000 (Invitrogen, Carlsbad, CA, USA), 20 ng wild-type (mβAhR/pcDNA3) or mutant AhR expression plasmids (in pcDNA3.1), 20 ng of mβARNT/pcDNA3 or pcDNA3.1(+) and 200 ng pGudLuc6.1 [17]. Twenty-four hours after transfection, cells were incubated with DMSO (0.1%, *v*/*v*), TCDD (10 nM final concentration) or IR (1 µM final concentration) for 18 to 22 h, followed by washing with PBS, lysis and measurement of luciferase activity in 50 µL aliquots as described above.

### 4.7. Statistical Analysis

Analysis of statistical significance of differences between experimental values was conducted using Two-Way ANOVA and EC_50_s and IC_50_s were calculated using nonlinear regression (three parameter) analysis in GraphPad Prism v.7 (La Jolla, CA, USA).

### 4.8. Molecular Modeling

The mAhR and hAhR PASB LBD structures were generated previously by homology modeling starting from the following sequences: UniProt id P30561 and residues 278–384 for mAhR; UniProt id P35869 and residues 284–390 for hAhR. Three ligand-bound X-ray structures of the HIF-2α protein (PDB ID: 3F1O, 3H7W and 3H82) were used as templates [35]. Four out of the one hundred models with the best DOPE score obtained by MODELLER [51] for each species were selected as representatives of the conformational variety of the modeled LBD by cluster analysis. Each model was refined by Molecular Mechanics (MM) energy minimization using MacroModel [52] maintaining a template ligand inside the binding cavity, as previously described [35]. The IR molecular structure was downloaded from PubChem (CID: 5359405) and prepared with LigPrep [53]. Two stereoisomers may exist at pH = 7 (cis and trans); in addition, a deprotonated form of IR is also considered to be present in water solution; all three forms were submitted to MM energy minimization and to the subsequent docking protocol. Molecular docking was carried out using Glide extra precision (XP) [54,55]. An ensemble docking approach, based on ligand docking to the four representative conformations of the modeled receptor, was used to include receptor flexibility. To take into account the induced-fit effects, a further optimization of the docking poses was performed using MacroModel [52]. Finally, the binding free energy (ΔG_bind_) of the obtained ligand-receptor complexes was calculated with the MM Generalized Born Surface Area (MM-GBSA) method [56] implemented in Prime MM-GBSA [57], which includes evaluation of the solvation contributions by an implicit solvent model. The obtained ΔG_bind_ values allowed the selection of the best pose among the ensemble. Visualization of the models was accomplished using PyMOL [58].

## Supplementary Materials

The following are available online at http://www.mdpi.com/1422-0067/19/9/2692/s1.
