# Supplementary material for: Comparative In Vitro and In Silico Analysis of the Selectivity of Indirubin as a Human Ah Receptor Agonist
Source: Int J Mol Sci. 2018 Sep 10;19(9):2692. doi: 10.3390/ijms19092692 (PMC6165432; doi:10.3390/ijms19092692)
Supplement: Supplementary file 1 [file ijms-19-02692-s001.pdf]

International Journal of
*Molecular Sciences*

MDPI

*Article*

# Comparative In Vitro and In Silico Analysis of the Selectivity of Indirubin as a Human Ah Receptor Agonist

**Samantha C. Faber [1], Anatoly A. Soshilov [1], Sara Giani Tagliabue [2], Laura Bonati [2] and Michael S. Denison [1,*]**

[1] Department of Environmental Toxicology, University of California, Davis, CA 95616, USA; scfaber@unc.edu (S.C.F.); soshilov@gmail.com (A.A.S.)
[2] Department of Earth and Environmental Sciences, University of Milano-Bicocca, Milan 20126, Italy; s.gianitagliabue@campus.unimib.it (S.G.T.); laura.bonati@unimib.it (L.B.)
* Correspondence: msdenison@ucdavis.edu; Tel.: +1-(530)-752-3879

Received: 7 August 2018; Accepted: 6 September 2018; Published: date

## Supplementary Data

Supplemental Figure S1. Mutations within the hAhR LBD that do not affect IR- or TCDD-selective AhR activation of the mAhR.

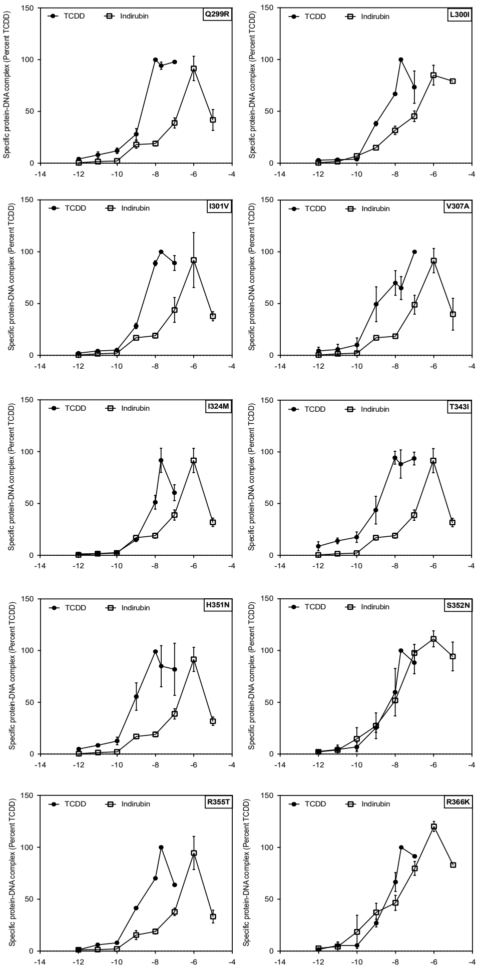


**Supplemental Figure S1**. Select mutations within the hAhR do not affect IR- or TCDD-selective AhR activation within the mAhR. *In vitro* synthesized mutant mAhRs were incubated in the presence of

solvent control DMSO (1%, vol/vol) or AhR agonists TCDD (0.001nM-100nM) or indirubin (0.001nM-10,000nM) for 2 h and analyzed by gel retardation assay. The amount of inducible protein-DNA complex at each TCDD or IR concentration was quantitated, and values normalized to the amount of complex formed with a maximal activating concentration of TCDD (20 nM). Values represent the mean ± SD of nine individual replicate analyses.

**S**upplemental Table S1. Relative potency ($EC_{50}$) of various AhR agonists to stimulate luciferase reporter gene expression in stably transfected mouse (H1L6.1c3) and human (HG2L6.1c1) hepatoma cells.

| | $EC_{50}$ (nM) ± SD | |
|---|---|---|
| Chemical | Human | Mouse |
| TCDD | 0.26 ± 0.07 | 0.027 ± 0.01 |
| TCDF | 4.10 ± 0.79 | 1.25 ± 0.65 |
| BNF | 36.80 ± 5.81 | 1.84 ± 1.14 |
| 3-MC | 14.56 ± 3.51 | 1.49 ± 0.01 |
| IR | 0.04 ± 0.02 | 15.60 ± 3.52 |
| ITE | 184.20 ± 42.09 | 22.32 ± 3.53 |
| FICZ | 51.97 ± 7.78 | 3.71 ± 0.85 |

**S**upplemental Table S1. Relative potency ($EC_{50}$) of various AhR agonists to stimulate luciferase reporter gene expression in stably transfected mouse (H1L6.1c3) and human (HG2L6.1c1) hepatoma cells. Cells were incubated with DMSO (1%, v/v), TCDD (0.1 nM-100 nM), TCDF (0.1 nM-100 nM), BNF (0.001 uM-10 uM), 3MC (0.001 uM-10 uM), IND (0.001 uM-10 uM), ITE (0.001 uM-10 uM), and FICZ (0.001 uM-10 uM) for 4 hr and luciferase activity determined as described in the Material and Methods. Luciferase activity (Relative Light Units (RLUs)) was normalized to the maximal induction observed with TCDD in each cell line. Values represent the mean ± SD of nine individual replicate analyses and EC50 values determined by nonlinear regression (three-parameter) analysis of graphical results shown in Figure 2.

Supplemental Table S2. Relative potency ($EC_{50}$) of TCDD and indirubin to stimulate transformation/DNA binding of wild-type mAhR and hAhR, mutant mAhRs and the mAhR-hAhRLBD chimera.

| | TCDD | Indirubin |
|---|---|---|
| AhR Construct | EC50 (nM) ± SD | |
| Wild-type constructs | | |
| mAhR | 2.48±0.54 | 14.80±4.19 |
| hAhR | 2.46±0.90 | 0.26±0.11 |
| Mutant mAhR constructs | | |
| mAhR-hAhRLBD | 1.64±0.68 | 5.05±2.07 |
| Q299R | 2.29±0.12 | 46.46±2.16 |
| L300I | 1.42±0.61 | 57.02±4.34 |
| I301V | 2.09±0.01 | 32.44±9.74 |
| V307A | 4.25±0.12 | 34.09±2.49 |
| I324M | 2.92±0.14 | 30.81±0.07 |
| H326Y | 0.62±0.52 | 1.00±0.42 |
| T343I | 1.79±0.12 | 21.26±1.66 |
| A349T | 3.86±1.06 | 0.08±0.01 |

| | | |
|---|---|---|
| H351N | 0.73±0.04 | 21.07±1.69 |
| S352N | 4.34±0.26 | 11.11±2.70 |
| R355T | 0.94±0.06 | 36.13±8.18 |
| A375V | 5.25±0.56 | 3.29±0.23 |
| Mutant hAhR constructs | | |
| T355A | 2.32±0.82 | 11.46±3.76 |
| V381A | 2.99±0.65 | 0.53±0.43 |
| Y332H | 0.59±0.6 | 33.65±0.73 |

Supplemental Table S2. Relative potency ($EC_{50}$) of TCDD and indirubin to stimulate transformation/DNA binding of wild-type mAhR and hAhR, mutant mAhRs and the mAhR-hAhRLBD chimera. I*n vitro* synthesized mutant mAhRs were incubated in the presence of solvent control DMSO (1%, v/v) or AhR agonists TCDD (0.001 nM-100 nM) or indirubin (0.001 nM-10,000 nM) for 2 h and DNA binding analyzed by the gel retardation assay as described in Materials and Methods. Values represent the mean ± SD of nine individual replicate analyses and $EC_{50}$ values determined by nonlinear regression (three-parameter) analysis and graphical depictions of the TCDD and indirubin DNA binding results are shown in Figures 3, 5 and S1.

**S**upplemental Table S3. Relative binding of indirubin to wild-type and mutant (A349T) mAhR and mAhR-hAhRLBD.

| mAhR Construct | IR $IC_{50}$ (nM) ± SD |
|---|---|
| mAhR | 17.67±1.66 |
| mAhR-hAhRLBD | 0.82±0.28 |
| A349T | 4.61±1.87 |

**S**upplemental Table S3. Relative binding of indirubin to wild-type and mutant (A349T) mAhR and mAhR-hAhRLBD. *In vitro* synthesized mAhR, mutant AhR, or mAhR-hAhRLBD chimeric protein was incubated in the presence of 2 nM $[^3H]$TCDD and increasing concentrations of IR for 30 min, and $[^3H]$TCDD binding was measured by the hydroxyapatite assay as described in Materials and Methods. Unprogrammed TNT lysate was used as a nonspecific binding control, and specific binding was calculated as a difference between the total and nonspecific reactions. Values represent the mean ± SD of nine individual replicate analyses based on nonlinear regression (three-parameter) analysis and graphical depictions of the indirubin competitive binding results are shown in Figure 7.

Supplemental Table S4. Mouse AhR PASB mutagenic primers.

| mAhR Mutant | Primer Sequence (5′-3′) |
|---|---|
| Q299R | tatagcccagaataagccgccctttggcatcacaa |
| L300I | tgtatagcccagaataatctgccctttggcatcac |
| I301V | tctgtatagcccagaacaagctgccctttggcat |
| V307A | ctcttgtgcacagctctgcttctgtatagcccaga |
| I324M | gattctgcacagtgaagcatgtctgcagcatggat |
| H326Y | tgggattctgcacaataaagtatgtctgcagcatggatgaac |
| T343I | gaagccggaaaactatcatgccactttctccagt |
| A349T | cctccagcgactgtgtttcgtaagaagccggaaaactgt |
| H351N | tccagcgactgtttttgcaagaagccggaaaactg |
| S352N | acctccagcgattgtgttttgcaagaagccgga |
| R355T | ggactggacccacgtccagcgactgtg |
| R366K | atgtaatctggtcttccatttttgtaaatcaagcgtgcattgg |
| A375V | tcagtggtctctgagtgacgatgatgtaatctggt |

Supplemental Table S4. Mouse AhR PASB mutagenic primer sequences were designed using Agilent QuikChange Primer Design (https://www.genomics.agilent.com/primerDesignProgram.jsp) and Mus musculus aryl-hydrocarbon receptor transcript variant mRNA (NM_013464.4, nucleotides 367-2784). Site-directed mutagenesis was carried out using the Agilent Technologies QuikChange Lightning Mutagenesis Kit and all constructs were verified by sequencing.
